# Thermolability of Syphilitic Antibodies

**Published:** 1919

**Authors:** 


**Thermolability of Syphilitic Antibodies.** By F. Gerard. *Comptes Rendus de la Société de Biologie.*

Comparative Wassermann reactions made on the same serum, using a non-heated serum and a serum heated to 56°, have demonstrated the existence of certain syphilitic antibodies which are completely thermolabile at 56°C. On the other hand, primary and secondary syphilis seem also thermolabile at 56°C.

In attempting a more rational dosage of the syphilitic antibodies by variations of the complement, two facts were revealed :

The antibody, acting as a catalyzer, can produce in minimum quantity very considerable deviations of complement in presence of a proper amount of antigen ; such as producing in 0.001 cc. of syphilitic serum from a guinea-pig the deviation of 1.25 cc. of complement to 1/10 in presence of 0.03 cc. antigen. All antibodies undergo a degradation at 56° C, though all do not present the same sensitiveness to heat. Generally a dose of antibodies capable of deviating an " X " quantity of complement cannot deviate more than two-thirds after being heated to 56° for half an hour — or the power is reduced by one-third.

At the same time, Gerard was not able to discover whether the greater thermolability of the antibodies corresponded to certain different periods of the disease. This thermolability is most variable and the antibody has its deviating power reduced by one-fifth, or sometimes as much as three-fourths.

Thermolability is traced also in other antibodies and particularly in natural anti-sheep hemolysins in man, which are very much degraded at 56° C. The anti-sheep hemolysins caused by injections of globules in rabbits are far less sensitive.

The heating test should be reduced to its minimum duration.

